# Repository corticotropin for Chronic Pulmonary Sarcoidosis

**DOI:** 10.1007/s00408-017-9994-4

**Published:** 2017-03-28

**Authors:** Robert P. Baughman, Nadera Sweiss, Ruth Keijsers, Surinder S. Birring, Ralph Shipley, Lesley Ann Saketkoo, Elyse E. Lower

**Affiliations:** 1 Department of Medicine, University of Cincinnati Medical Center, 1001 Holmes, Eden Ave, Cincinnati, OH 45267 USA; 2 Department of Medicine, University of Illinois Chicago, Chicago, IL USA; 3 Department of Nuclear Medicine, St. Antonius Ziekenhuis Nieuwegein, Nieuwegein, The Netherlands; 4 Division of Asthma, Allergy and Lung Biology, King’s College London, King’s Health Partners, London, UK; 5 Department of Radiology, University of Cincinnati Medical Center, Cincinnati, OH USA; 6 Department of Medicine, Tulane University, New Orleans, LA USA

**Keywords:** Sarcoidosis, Repository corticotrophin, Acthar gel, PET scan, Quality of life

## Abstract

**Purpose:**

The dose of repository corticotropin (RCI) and need for a loading dose in sarcoidosis patients receiving chronic corticosteroids are unclear. We performed a single-blind prospective study, comparing two doses of RCI in sarcoidosis.

**Methods:**

Chronic pulmonary sarcoidosis patients receiving prednisone therapy with deterioration by 5% in FVC in the previous year were studied. RCI was administered subcutaneously at a loading dose of 80 units RCI for 10 days. Patients were randomized at day 14 to receive either 40- or 80-unit RCI twice a week. The dose of prednisone was modified by the clinician who was blinded to the patient’s dosage of RCI.

**Results:**

Sixteen patients completed the full 24 weeks of the study. At week 24, there was a decrease in the dose of prednisone, and improvements in DLCO, King’s Sarcoidosis Questionnaire health status and fatigue score. There was no significant change in FVC % predicted. For the PET scan, there was a significant fall in the standard uptake value (SUV) of the lung lesions. Only 3/8 patients remained on 80 units RCI for full 24 weeks. There was no significant difference in the response to therapy for those treated with 40- versus 80-unit RCI.

**Conclusions:**

Repository corticotropin treatment was prednisone-sparing and associated with significant improvement in DLCO, PET scan, and patient-reported outcome measures. A dose of 40-unit RCI twice a week was as effective as 80-unit RCI and was better tolerated.

## Introduction

Long-term treatment with anti-inflammatory drugs such as corticosteroids has been the mainstay of treatment of symptomatic pulmonary sarcoidosis [1]. However, significant toxicity is often encountered in patients receiving prolonged dosage [2]. In patients who have deteriorated while having their prednisone withdrawn, several alternatives have been investigated. These include oral cytotoxic agents such as methotrexate and azathioprine [3] as well as the biologic agent infliximab [4].

In some conditions, the use of repository corticotropin (RCI) has been found to be similar or more effective than oral corticosteroids, with less toxicity [5–7]. A recent report demonstrated steroid sparing and, in some cases, improvement in patients with advanced sarcoidosis [8]. However, the dose of RCI and need for a loading dose in patients receiving chronic corticosteroids were unclear. Since toxicity may be dose-dependent and the drug is expensive, a reduction of dose may have significant impact on its use. We performed a study comparing two doses of RCI in advanced pulmonary sarcoidosis patient receiving prednisone. The primary endpoint of the study was prednisone-sparing effect of RCI. We also evaluated the effect of RCI on pulmonary function, chest imaging, and health-related quality of life in advanced pulmonary sarcoidosis.

## Methods

This was a multi-center single-blind trial of patients with chronic pulmonary sarcoidosis patients. All patient met ATS criteria [1] for diagnosis and were on a stable dose of 5 mg or more prednisone for at least 3 months. Patients had deterioration of their pulmonary disease in the previous year. Patients were excluded if they had received anti-TNF antibody (e.g., infliximab, adalimumab) in prior 6 months or were receiving treatment for sarcoidosis-associated pulmonary hypertension. The study protocol was approved by the local Institutional Review Board and listed in Clinical Trials as NCT02188017. All information was recorded using remote data capture (RedCAP) [9] and included some forms from the RedCAP shared library [10].

All eligible patients were scheduled to receive a loading dose of 80 Units RCI subcutaneously (SQ) once a day for 10 days. Per protocol, patients who complained of intolerance to daily treatments were instructed to stop the loading dose at less than 10 days. Fourteen days after starting the study (4 days after the last scheduled daily dose), the subjects were randomized 1:1 to receive either 40- or 80-unit RCI twice a week for 22 more weeks. If the patient complained of side effects from treatment, they were instructed to halve their dose of corticotrophin. The investigators were blinded to the dose the patient was receiving throughout the study.

Patients were seen at 2, 7, 11, and 24 weeks after starting on RCI. At each visit, they were assessed for modification of their prednisone dose based on previously established algorithm [2]. If patients were felt to be improved, the dose of prednisone was halved. If they had been stable for two visits in a row (including the week 0 visit), the dose was halved. If the patient was clinically worse, the prednisone dose was doubled. If the patient was stable for only one visit, there was no change in prednisone dosage. The dose could also be reduced for prednisone toxicity. The clinician was blinded to the dose of RCI that the patient was receiving.

At weeks 0, 7, and 24, patients underwent history and physical examination, completed various questionnaires including the King’s Sarcoidosis Questionnaire (KSQ) [11], Saint George Respiratory Questionnaire (SGRQ) [12], and Fatigue Assessment Scale (FAS) [13], and performed spirometry and 6-min walk testing [14]. At week 0 and 24, patients had their diffusion of lung of carbon monoxide (DLCO) measured. Predicted values for spirometry and DLCO were calculated [15, 16].

High-resolution computer tomography (HRCT) and [18] F-fluorodeoxyglucose positron emission tomography (FDG PET) scanning were performed initially and after 24 weeks of therapy. The paired HRCT scans were compared using a five-point Likert scale (much worse to much better) by a single reader (RS). The FDG PET scan was centrally read by one reader (RK) blinded to the treatment regimen. For the FDG PET scan, the highest standardized uptake value (SUV) for the thorax and for any other region was noted after the first scan. The SUVs for those same areas were then determined at the 24-week scan.

We prospectively examined the toxicity by regularly measuring hemoglobin A1C, serum glucose, blood pressure, and weight. Patients also completed a steroid toxicity questionnaire [2].

### Statistics

The study was analyzed as an intention to treat analysis, and at week 7, we compared those patients still receiving 80 units RCI versus those receiving 40-unit RCI. Comparisons were made using Mann–Whitney U test and Wilcoxon test for paired samples. A *p* value of less than 0.05 was considered significant.

## Results

### CONSORT

**Fig. 1:**
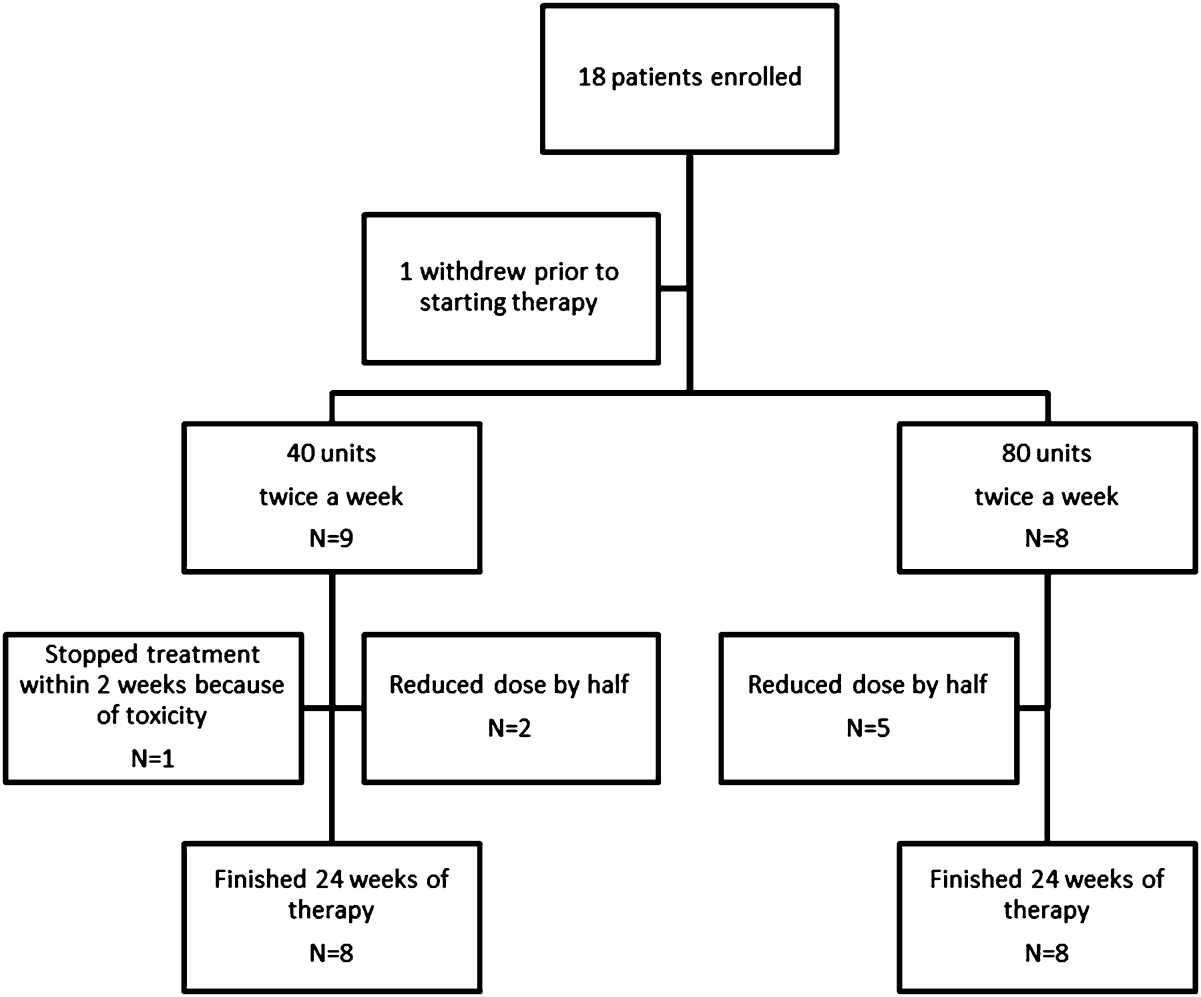
CONSORT flow sheet of patients enrolled in study. Seven patients reduced their treatment dose by half. Sixteen patients (eight for each treatment arm) completed the 24 weeks of study

Eighteen patients were enrolled into the study. Figure 1 shows the CONSORT [17] flow sheet of outcome of all patients in the study. One patient withdrew before taking any therapy. Eight patients complained of one or more of the following: jitteriness (six patients), headache (three patients), edema (two patients), and nausea (one patient). One patient had the dose held because of an episode of herpes zoster. The treatment was reinstituted 7 days later. Over the course of the study, one patient withdrew during the initial loading period of 80 units RCI a day for 10 days. An additional five patients stopped the loading dose between 7 and 9 days. These five patients remained in the study and began the assigned dose of either 40- or 80-unit RCI at day fourteen. Patients could reduce the dose by half for perceived problems with drug treatment. During the 24 weeks of the study, seven patients reduced their dose by half (two of eight in the 40-unit RCI arm versus five of eight in the 80-unit RCI arm, *p* > 0.05). At week 7, six patients were receiving 80-unit RCI while ten patients were receiving 40-unit RCI.

### Demographics

Table 1 shows the demographic features of the sixteen patients who completed the 24-week study. There was no significant difference for the two patients who did not participate in the study for more than 2 weeks and they were not further analyzed. There were no differences between those assigned to 40 versus 80 units of RCI in terms of age, race, sex, or initial pulmonary function tests. Table 2 summarizes the current and past immunosuppressive therapy used for the two groups, with no difference between those assigned 40- versus 80-unit RCI. Since there was no significant difference in the features of those assigned 40 versus 80 units of RCI, we also analyzed the effect of RCI therapy for the whole group.

**Table 1:** Demographics of patients

	40-unit RCI	80-unit RCI
Number	8	8
Age, years	58 (49–68)	59 (35–60)
Male:female	3:5	4:4
African American: caucasian	4:4	3:5
FEV-1, L	1.59 (1.17–2.28)	1.78 (0.83–2.30)
FEV-1% predicted	52 (36–79)%	44.4 (36–77)%
FVC, L	2.42 (1.48–3.20)	2.51 (1.34–3.55)
FVC % predicted	68 (41–83)%	66 (48–71)%
FEV-1/FVC %	71 (49–85)%	64 (48–90)%
DLCO mL CO (STPD)/min/mmHg	13.75 (10.13–18.26)	14.50 (7.80-22.88)
DLCO % predicted	55 (23–73)%	61 (29–81)%
Organ involvement		
Skin	4	2
Sinus	3	1
Eyes	0	2
Liver	0	1
Spleen	1	1
Neurologic	0	1
Extra-thoracic nodes	1	1
Parotid	1	0
Abnormal calcium metabolism	2	0

**Table 2:** Anti-inflammatory therapy used for sarcoidosis

Drug	40-unit RCI	80-unit RCI
Prednisone	8/0^a^	8/0
Methotrexate	2/4	0/2
Azathioprine	1/1	0/2
Leflunomide	1/1	0/1
Mycophenolate	0/1	0/0
Hydroxychloroquine	1/5	0/2
Infliximab	0/2	0/2
Adalimumab	0/1	0/2

^a^Current/past usage

### Corticosteroid reduction

**Fig. 2:**
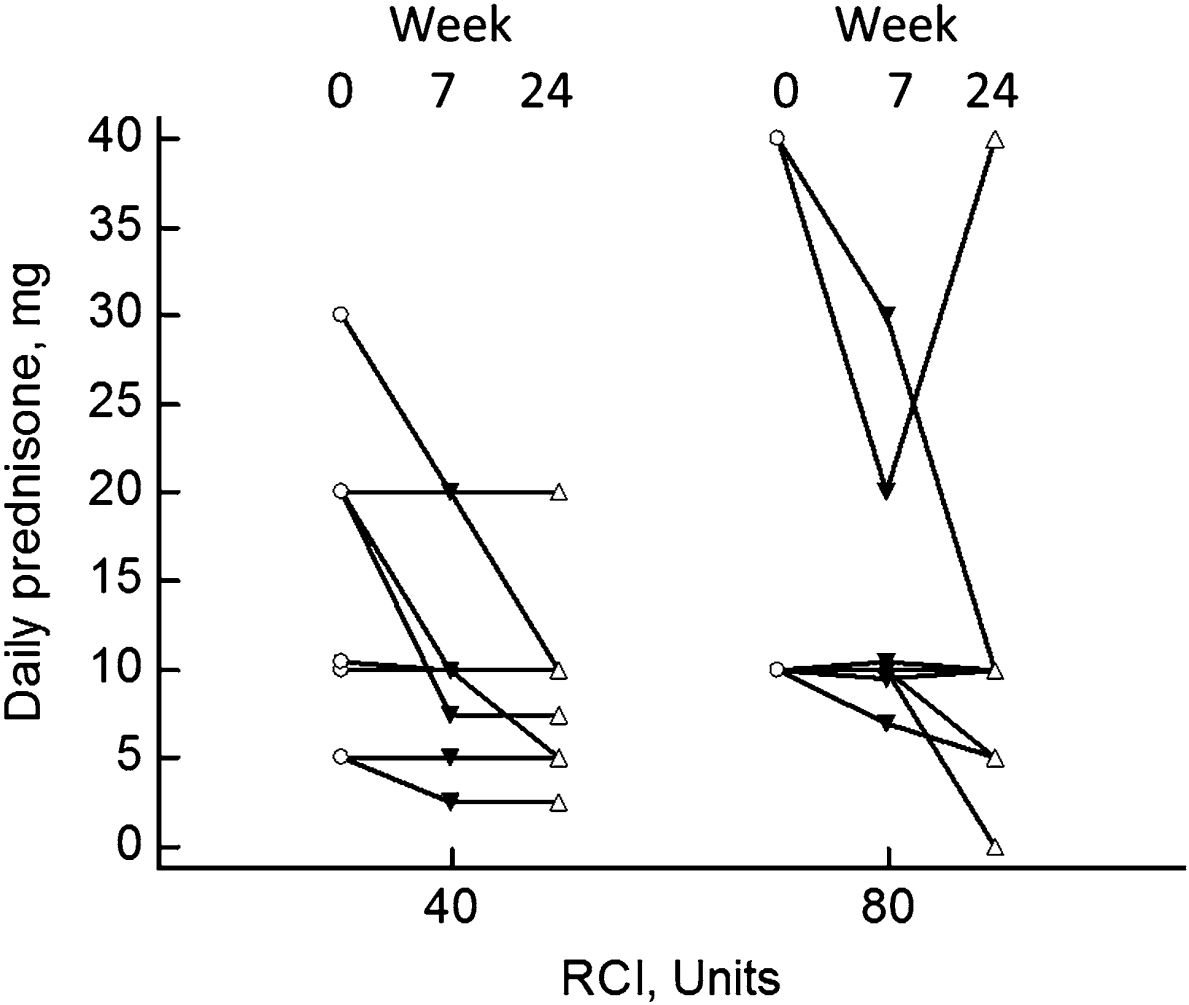
Daily prednisone dose initially and at weeks 7 and 24. There was a significant fall in the prednisone dose by week 7 (*p* = 0.00156) which persisted through week 24 (*p* = 0.0078). The values for those assigned to 40- versus 80-unit RCI are shown. There was no difference between the two groups

All patients were receiving prednisone at the start of the study. Figure 2 demonstrates the daily prednisone dose initially and at weeks 7 and 24 for those assigned the 40 versus 80 units. At week 7, there was no significant difference in reduction of prednisone dosage for those receiving 80 units RCI (median 0 mg, range 0 to −20 mg) versus those receiving 40-unit RCI (median −2.75 mg, range 0 to −12.5 mg, *p* > 0.05). We then examined the effect of RCI at any dose. Compared to the initial dosage of prednisone, there was a significant fall in the prednisone dosage at 7 weeks (*p* = 0.0156) and 24 weeks (*p* = 0.0078). There was no significant difference between the prednisone dose at 7 versus 24 weeks. There was no significant difference between the 40- and 80-unit treatment groups.

### Lung function

After seven weeks, there was no significant difference in the change in the FVC % predicted for those receiving 80-unit RCI (median 1.0%, range −9.0 to 6%) versus those receiving 40-unit RCI (median −3.0%, range −6.0 to 11.0%, *p* > 0.05). The changes in pulmonary function testing during RCI therapy at 7 and 24 weeks for all 16 patients are summarized in Table 3. Compared to initial values, there was no significant difference in the FVC or FEV-1. Five patients had a 5% or greater increase of the absolute FVC % predicted.

**Table 3:** Pulmonary function testing before and after 7 and 24 weeks of therapy

	Initial	Week 7	Week 24
FVC, liters	2.42 (1.34–3.55)^a^	2.30 (1.08–3.87)	2.41 (1.15–3.65)
Change in FVC from Week 0		−0.08 (−0.31, +0.40)	−0.05 (−0.28, +0.19)
FVC % predicted	66 (41–83)	69 (39–86)	69 (40–91)
Change in FVC % predicted from week 0, %		−2.4 (−9.0, 11.0)	−2.0 (−9.0,+16.0)
FEV-1, L	1.63 (0.83–2.3)	1.58 (0.74–2.69)	1.55 (0.69–2.35)
Change in FEV-1 from week 0, L		−0.02 (−0.21, +0.41)	−0.12 (−0.48, +0.13)
FEV-1% predicted	46 (36–79)	48 (30–94)	46 (24–95)
Change in FEV-1% predicted from week 0, %		−0.5 (−10.0, +15)	−3.0 (−17, +16.0)
FEV-1/FVC	70 (48–90)	68 (47–87)	62 (41–86)
Change in FEV-1/FVC from week 0, %		0.01 (−0.04, +0.07)	−0.02 (−0.31, +0.04)
DLCO mm Hg	14 (7.8–22.88)	15.54 (7.7–23.38)	15.28 (5.8–24.14)
Change in DLCO from week 0			0.64 (−5.01, +6.28)
DLCO % predicted	59 (23–81)	58 (33–84)	62 (24–99)^b^
Change in DLCO % predicted from week 0, %			9.5 (−14.0, +27)
6MWD, m	381 (152–610)	408 (183–655)	388 (152–701)
Change in 6MWD, m		0 (−69 to 175)	0 (−137 to 297)

^a^Median (range)

^b^Differs from week 0, *p* = 0.0419

**Fig. 3:**
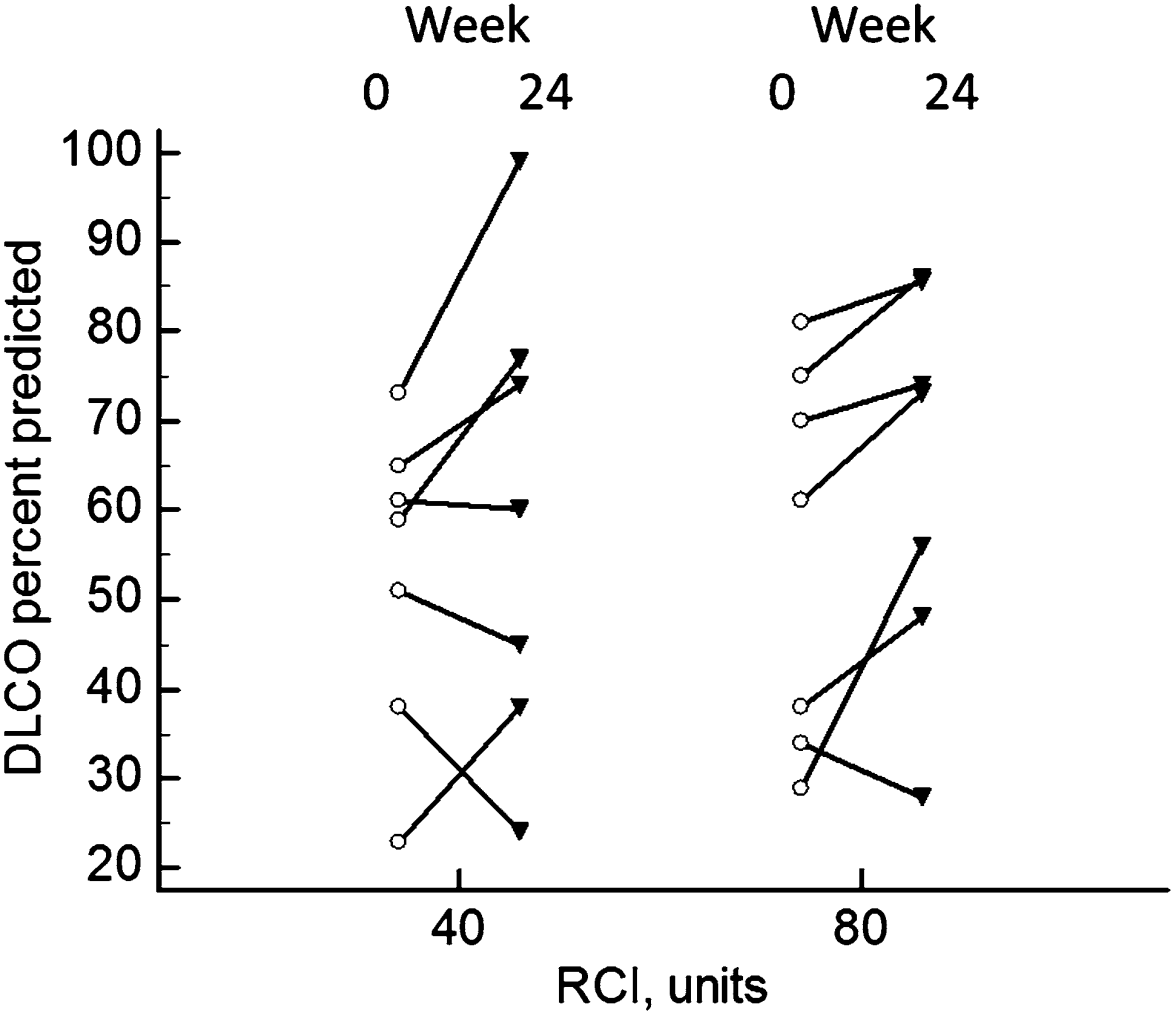
DLCO percent predicted at weeks 0 and 24. For all patients, there was a significant rise in DLCO at week 24 (*p* = 0.0419). The values for those assigned to 40 versus 80 units RCI are shown. There was no difference between the two groups

Fourteen patients had their DLCO measured prior to and after 24 weeks of therapy. There was a significant rise in the DLCO percent predicted after 24 weeks of therapy (Fig. 3, *p* = 0.0419). There was no significant difference in the 6MWD at either 7 or 24 weeks. There was no significant difference in the change in DLCO for the 40 versus 80 units RCI group.

### Imaging

Of the 16 paired HRCT studies, one was scored as somewhat worse, nine as same, and five (31%) as somewhat better at week 24. Table 4 summarizes the changes in pulmonary function data of these patients. The change in DLCO percent predicted was significantly higher for the somewhat better group versus those with no improvement in HRCT (same or somewhat worse, *p* = 0.0237).

**Table 4:** HRCT score versus change in pulmonary function after 24 weeks of therapy

	Somewhat worse	Same	Somewhat better
Number of patients	1	9	5
FVC, L	−0.11	−0.04^a^ (−0.28, 0.19)	−0.11 (−0.19, 0.14)
FVC percent predicted, %	−3	−1 (-9, 6)	−2 (-5, 16)
FEV-1, L	−0.18	−0.11 (−0.48, 0.13)	−0.17 (−0.24, 0.07)
FEV-1 percent predicted, %	−7	−1 (−17, 6)	−3 (−10, 16)
FEV1/FVC ratio, %	−0.04	−0.01 (−0.31, 0.04)	−0.02 (−0.09, 0.031)
DLCO, mL CO (STPD)/min/mmHg	1.17	−0.22 (−5.01, 6.28)	2.905 (−1.66. 3.8)
DLCO percent predicted, %^b^	10	4 (−14, 26)	16.5 (11, 27)
6-min walk distance, m	0	−3.81 (−137, 297)	61.9 (0, 260)

^a^Median (range)

^b^Significantly different from same, *p* = 0.0308

**Fig. 4:**
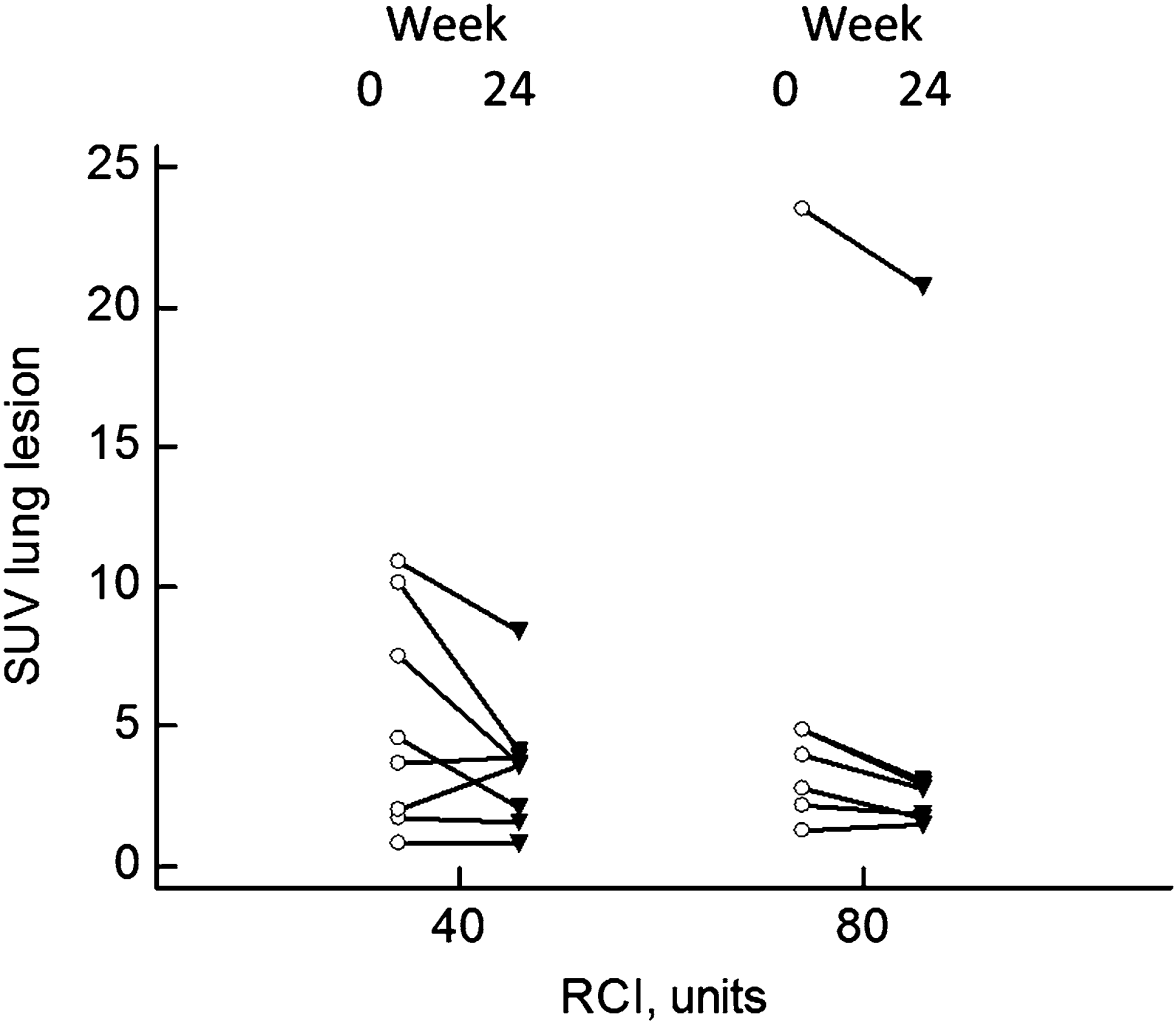
Change in SUV of highest lung lesion before and after 24 weeks of RCI therapy. Overall, the SUV fell from median 4.0 to 2.9 (*p* = 0.0085). The values for those assigned to 40 versus 80 units RCI are shown. There was no difference between the two groups

Fifteen patients had technically adequate PET scans before and after 24 weeks of RCI therapy. There was a significant fall in the SUV of the highest lung lesion from median 4.0 (range 0.8–23.5) to 2.9 (range 0.8–20.7, *p* = 0.0085). Figure 4 shows the individual values for each patient for those assigned to either 40 or 80 units RCI. There was no correlation between the PET scan results and the change in DLCO, FVC percent predicted, or dose of prednisone during the 24 weeks of the study.

### Patient-reported outcomes

**Fig. 5:**
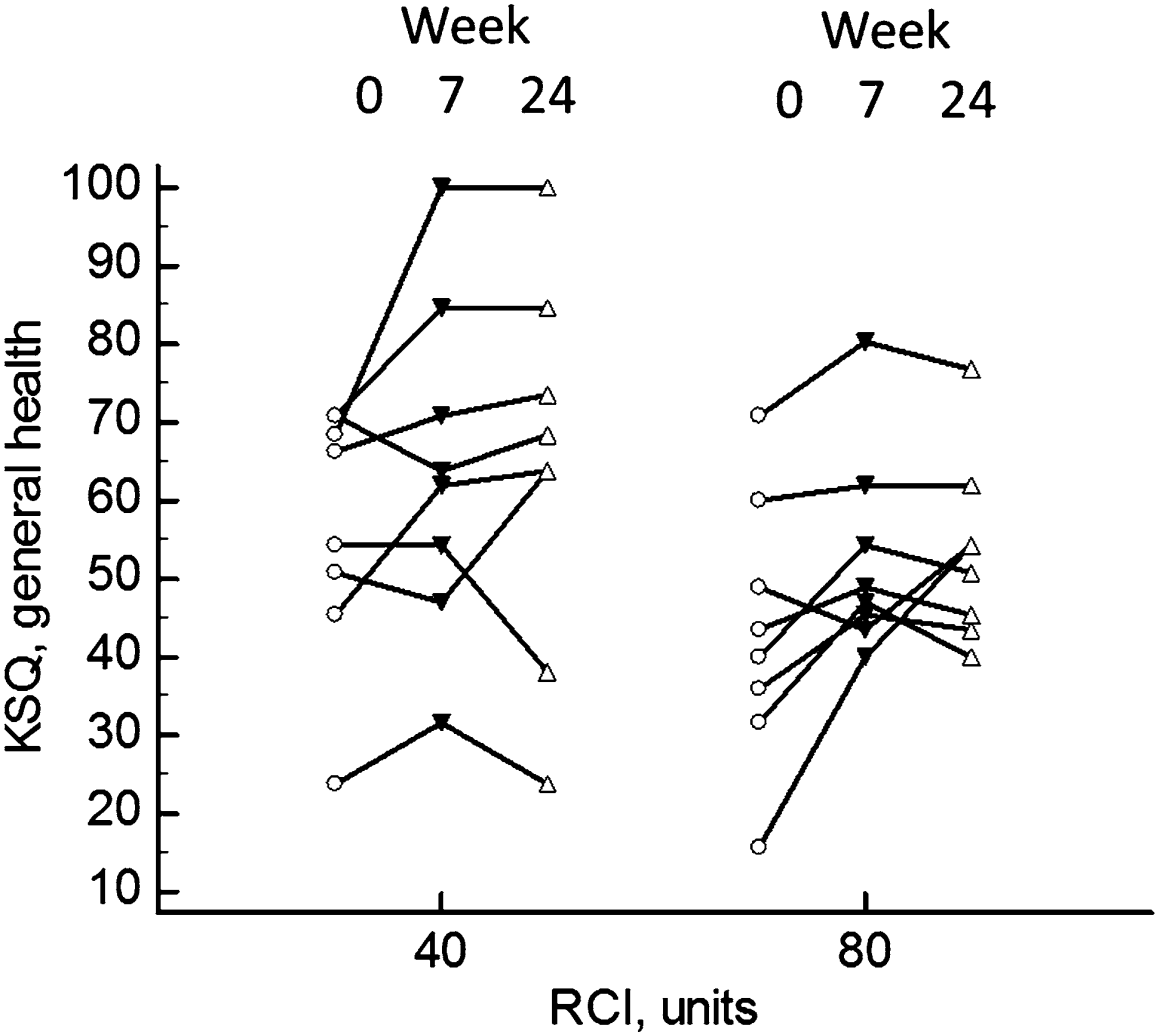
King’s Sarcoidosis questionnaire (KSQ) general health at weeks 0, 7, and 24. Overall, there was a significant rise (better health) in score at week 7 (*p* = 0.0043) and week 24 (*p* = 0.0084). The values for those assigned to 40 versus 80 units RCI are shown. There was no difference between the two groups

Table 5 summarizes the changes in quality of life using three instruments. There were significant differences in the KSQ for several domains, including general health status (GHS), general health status lung (GHS lung), and lung. At 7 weeks, there was no significant difference in the improvement in GHS between those receiving 80 units RCI (median 7.4, range −5.4 to 15.4) versus those receiving 40 units RCI (median 10.8, range −7.0 to 31.6, *p* > 0.05). For all patients, there was a significant improvement (rise) in GHS at weeks 7 and 24 versus week 0 (Fig. 5). There was no significant difference in the change in GHS for those assigned to 40 versus 80 units RCI. Neither the SGRQ total or any of its three components changed significantly during the study. There was a significant fall (less fatigue) in the FAS score at week 24 (*p* = 0.0067). At week 7, ten patients had a four-point or greater drop in their FAS score. By week 24, eight patients still had a four-point or greater drop in FAS from initial values.

**Table 5:** Quality-of-life changes during therapy

	Initial	Week 7	Week 24
Kings sarcoidosis questionnaire			
GHS	49.8 (15.9–70.9)^a^	54.3 (31.7–100)^b^	58.1 (23.8–100)^c^
GHS Lung	51.5 (41.0–68.0)	57.8 (47.7–100)^d^	56.9 (44.3–100)^f^
Lung	42.8 (22.3–61.0)	54.4 (33.6–100)^e^	49.6 (37.2–100)
Saint George respiratory questionnaire			
SGRQ activity	74.44 (35.24–92.51)	72.82 (41.63–92.51)	72.82 (47.69–92.51)
SGRQ impacts	36.68 (13.75–70.34)	40.92 (19.34–66.55)	42.52 (17.19–66.7)
SGRQ symptoms	53.465 (17.24–92.65)	57.98 (32.51–90.26)	46.28 (7.63–85.84)
SGRQ total	51.49 (21.89–77.21)	54.88 (34.1–70.31)	54.13 (25.4–71.1)
Fatigue assessment scale			
FAS	28 (15–46)	26 (10–37)	22 (11–42)^g^

^a^Median (range). *GHS* general health status

^b^Compared to week 0, *p* = 0.0043

^c^Compared to week 0, *p* = 0.0084

^d^Compared to week 0, *p* = 0.0034

^e^Compared to week 0, *p* = 0.0067

^f^Compared to week 0, *p* = 0.0107

^g^Compared to week 0, *p* = 0.0067

### Adverse events

All patients completed a previously described prednisone toxicity questionnaire [2]. There was no significant difference in the reported toxicity including changes in moodiness, appetite, or bruising. Over the 24 weeks of the study, there was no significant change in weight. Six of the patients had an elevated hemoglobin A1C at time of study entry. None of these six patient’s hemoglobin A1C fell into normal range by the end of the 24-week study. There were no changes in the patient’s diabetic or hypertensive medications during the course of the study. There was no difference for those in the 40- versus 80-unit RCI group.

## Discussion

This was a prospective single-blind study comparing two doses of RCI in patients advanced pulmonary sarcoidosis. After 7 weeks, there was no significant difference between those receiving 80 versus 40 units of RCI twice a week. Over the next 17 weeks, the dose was adjusted in many patients. When we analyzed the effect of RCI therapy for all patients, regardless of dose that the patient received, RCI therapy was associated with a significant reduction in the dosage of prednisone. Despite withdrawal of prednisone, patients had a significant improvement in their DLCO. In addition, there was a significant improvement in the health-related quality of life while in the study, including lung health and reduction of fatigue.

Glucocorticoids have been the cornerstone of treatment of symptomatic sarcoidosis despite the limited number of randomized trials. The initial reports supporting use of glucocorticoids included patients also treated with RCI as a method to stimulate glucocorticoid release [18]. For many years, RCI use was extremely rare in sarcoidosis due to the cost and presumption that glucocorticoid release was their only mechanism of action. Melanocortin receptors have been identified on other cells than the adrenal cortex, including inflammatory cells. Studies in infantile spasticity, renal disease, and multiple sclerosis have suggested that the stimulation of these melanocortin receptors may be an important mechanism of action [19–22]. Recent experience with using RCI for advanced sarcoidosis has suggested benefit for some patients with sarcoidosis [8, 23].

The previous trials left answered some questions regarding dose and frequency of administration. Long-term prednisone use is associated with reduced response to ACTH [24]. In one study of RCI for nephrotic disease, 80-unit RCI twice a week was more effective but more toxic that 40-unit RCI twice a week [22]. In the current study, five patients stopped the loading schedule at days seven through nine due to RCI toxicity. There seemed no benefit for a prolonged loading dose.

For patients assigned to 80-unit RCI twice a week, five of eight reduced their dose to 40-unit RCI twice a week, while only two of eight assigned to 40-unit RCI twice a week reduced their dose. There was no difference in the response for those who were assigned to 80- or 40-unit RCI twice a week. At week 7, there were six patients receiving 80 units of RCI versus ten patients receiving 40 units of RCI. There was no significant difference between the two doses in the reduction of prednisone dosage, changes in FVC % predicted, or the quality of life of the patients. When we compared those initially assigned to either 80 or 40 units of RCI, there were no differences at either 7 or 24 weeks of the study.

The primary endpoint of this trial was the prednisone-sparing effect of RCI. Treatment with RCI led to a significant reduction in prednisone dose within 7 weeks and this persisted for the full 24 weeks of the study (Fig. 2). It has been previously noted that RCI was associated with reduction of prednisone in most patients who remained on drug for more than 3 months [8]. We were unable to demonstrate that the reduction in prednisone dosage during the short course of this study was associated with lessening of the toxicities associated with high-dose prednisone such as weight gain and diabetes. However, patients reported an improved quality of life while receiving RCI.

A change in FVC percent predicted is a commonly reported endpoint in clinical trials of sarcoidosis [25, 26]. A change of 5% or greater has been noted in placebo-controlled trials of glucocorticoids [27, 28] and a subset of those treated with infliximab [4]. In the current study, a 5% or greater change in FVC percent predicted at 24 weeks was seen in four patients. There were eight patients who had a ten percent or greater rise in the DLCO percent predicted at week 24. Nine of 16 (56%) of patients had either a significant rise in DLCO or FVC percent predicted.

This study examined two lung imaging methods. High-resolution computer tomography (HRCT) has not been routinely studied in sarcoidosis treatment trials. A scoring system has been used to assess lung and lymph node involvement, but does not provide a summation of response to treatment [29]. We used a scoring system comparing the HRCT before and after 24 weeks of therapy. This scoring system is similar to that used to score changes of routine chest X-ray with treatment [30–32]. Five patients had a somewhat better HRCT after treatment and had a significantly greater improvement of their DLCO after treatment compared to those without improvement of their HRCT.

The reduction of the maximal SUV of a lung lesion has been reported for patients treated with infliximab [33]. In the current study, we also observed a significant fall in SUV lung and extra-thoracic lesions for most patients. This change in SUV occurred despite the reduction of prednisone dose.

In the current study, we examined prospectively three patient-reported outcomes. The KSQ was developed to assess several components [11]. We focused on three major components: general health status, lung disease, and combined general health status and lung. All three of these components improved significantly during treatment with RCI. The improvement averaged more than four points, which has been determined to be the minimally clinically important difference (MCID) [11]. The fatigue assessment scale (FAS) is a widely used instrument to assess sarcoidosis-associated fatigue [13]. The FAS score has been shown to improve with therapy with neurostimulants [34, 35] and physical training [36]. A change in FAS of 4 or more points was reported as a (MCID) [37] and was reported in half of the patients. There was no significant difference in the SGRQ during this study.

We prospectively collected adverse events associated with corticotopin use. In the current study, there was no significant change in weight during the course of the study. There was also no change in hemoglobin A1c levels or treatment for diabetes or hypertension. Eight patients complained of anxiety and fluid retention on the day of drug administration. Many of these occurred during the daily loading doses. One patient withdrew from the study, the others stopped the loading dose, and/or reduced their dose by 50% and remained in the study.

There are several limitations to the current study. Many patients did not complete the proposed ten-day loading dose due to adverse events reported by the patients. While discontinuation of loading dose was part of the protocol, the high number of patients who did not complete all 10 days made it difficult to identify if there was any benefit from taking a loading dose. Our impression was that the loading dose added no benefit but enhanced toxicity. For the maintenance phase of the study, the protocol was designed to allow patients halve their dosage of RCI. By 24 weeks, only two patients were receiving 80 units RCI. Therefore, we could not analyze the effect of 80-unit RCI on steroid sparing or lung function at week 24. However, we did have sufficient number of patients to compare 40 versus 80 units at 7 weeks. Since there was no significant difference at 7 weeks, we chose to analyze the effect of any dose of RCI on steroid sparing, lung function, or quality of life. For the group, there were significant changes in some of these parameters. The study was designed to examine the effect of RCI on toxicity and prednisone sparing. Since there was no placebo group, we cannot be sure that reduction in prednisone dose was not due to effect of repeat study visits and effort to reduce prednisone. The reduction of prednisone dose may have also been due to stimulation of glucocorticoid release. The small number of patients limits our ability to comment on the effect of RCI on pulmonary function. Also the study was a single-blind study. The positive results of this study may help in identifying which patients may benefit from RCI. In addition, the study may prove useful information for designing future trials comparing RCI to placebo.

In conclusion, we found that RCI was associated with a significant reduction of prednisone dosage. The reduction in prednisone dose may have been related to the glucocorticoid effect of RCI. Treatment with RCI was associated with a significant improvement in pulmonary function and chest imaging. RCI therapy was associated with improved health-related quality of life and less fatigue. The use of 40 units of RCI twice a week was not inferior to 80 units of RCI.
